# Efficacy and Safety of Anti-Respiratory Syncytial Virus Monoclonal Antibody Nirsevimab in Neonates: A Real-World Monocentric Study

**DOI:** 10.3390/vaccines13080838

**Published:** 2025-08-06

**Authors:** Maria Costantino, Mariagrazia Bathilde Marongiu, Maria Grazia Corbo, Anna Maria Della Corte, Anna Rita Frascogna, Angela Plantulli, Federica Campana, Luigi Fortino, Emanuela Santoro, Emilia Anna Vozzella, Walter Longanella, Giovanni Boccia, Amelia Filippelli, Francesco De Caro

**Affiliations:** 1 Department of Medicine, Surgery, and Dentistry, University of Salerno, 84081 Baronissi, Italy; a.dellacorte11@studenti.unisa.it (A.M.D.C.); f.campana@studenti.unisa.it (F.C.); esantoro@unisa.it (E.S.); gboccia@unisa.it (G.B.); afilippelli@unisa.it (A.F.); fdecaro@unisa.it (F.D.C.); 2 University Hospital “San Giovanni di Dio e Ruggi d’Aragona”, 84131 Salerno, Italy; maria.corbo@sangiovannieruggi.it (M.G.C.); annarita.frascogna@sangiovannieruggi.it (A.R.F.); angela.plantulli@sangiovannieruggi.it (A.P.); luigi.fortino@sangiovannieruggi.it (L.F.); direzione.sanitaria@sangiovannieruggi.it (E.A.V.); walter.longanella@sangiovannieruggi.it (W.L.); 3 Department of Women, Child and General and Specialized Surgery, University “Luigi Vanvitelli”, 80138 Naples, Italy; mariagraziabathilde.marongiu@studenti.unicampania.it

**Keywords:** Respiratory Syncytial Virus, newborns, AEFIs, nirsevimab

## Abstract

Background: RSV remains a leading cause of infant hospitalization worldwide, and the recently approved nirsevimab could represent an effective and safe prophylactic strategy to prevent severe infections in the general neonatal population. Objectives: We conducted a retrospective observational monocentric pilot study in a mixed preterm/term birth cohort to add real-world evidence of the efficacy and safety of nirsevimab in preventing severe RSV infection. Methods: We included a total of 2035 consecutive infants admitted to the Neonatal Unit, University Hospital “San Giovanni di Dio e Ruggi d’Aragona”, Salerno, Italy, from November 2024 to April 2025. We evaluated 30-day safety profiles and season-wide RSV infection rates, and the outcomes were also compared to newborns’ birth rate in the two previous seasons (2022–2023 and 2023–2024). Results: After the introduction of nirsevimab, a lower RSV infection rate was reported compared to previous seasons, and no adverse effects were observed. Compared to previous seasons, the clinical outcomes were more favorable, as only one unvaccinated neonate with RSV infection required invasive ventilation. Conclusions: In this real-world analysis, we demonstrated a good short-term safety profile of nirsevimab, as well as a potentially high efficacy in the general neonatal population with lower RSV infection incidence. However, future studies are needed to better assess its long-term safety and season-wide efficacy.

## 1. Introduction

Among recent advances in RSV infection prevention, nirsevimab is a long-acting MoAb with a half-life of 5 months, offering seasonal protection with a single shot [1,2,3,4]. Indeed, RSV prophylaxis with nirsevimab reduces hospitalizations in both healthy and preterm infants [5,6]. RSV infection affects newborns worldwide, accounting for ~3.6 million hospitalizations and >100,000 deaths per year, 95% of which occur in low- and middle-income regions [7,8]. RSV mostly affects healthy newborns and infants, as 80–90% of hospitalized children are full-term infants without known risk factors, and primary infection often requires corticosteroids, ventilatory support, and/or enteral nutrition [9,10,11,12]. The presence of just one risk factor, such as preterm birth, bronchopulmonary dysplasia, congenital heart disease, chronic respiratory conditions, or immunocompromised status, leads to longer hospitalizations and worse outcomes.

Several countries, including France, Spain, the United States, and Luxembourg, have implemented prevention plans to reduce RSV incidence, showing a significant reduction in hospitalizations and intensive care unit admissions for RSV bronchiolitis [9,13]. In Italy, RSV circulates seasonally, emerging in October, peaking between December and February, and declining by March–April [14,15,16]. In Italy, the RSV prophylaxis campaign has been initiated in 2023–2024 in the Valle d’Aosta region, which reported zero hospitalizations for RSV-caused lower respiratory tract diseases in newborns who received it [17]. Based on this pilot program, starting from November 2024, nirsevimab has been introduced in routine clinical practice, but real-life data on its efficacy and safety are still limited, and robust pharmacoeconomic evidence to include nirsevimab in the Italian National Vaccination Plan is needed. Moreover, vaccination hesitancy is another significant barrier to effective infectious disease prevention [18,19,20], and clear evidence of its efficacy and safety can help alleviate parental concerns and highlight economic benefits.

In this retrospective, observational, monocentric, real-world pilot study, we investigated the efficacy and safety of nirsevimab in a cohort of term and preterm newborns during the 2024–2025 RSV season, and the results were compared to the data obtained from the two preceding epidemic seasons.

## 2. Materials and Methods

### 2.1. Population and Study Design

In this retrospective, monocentric, real-world pilot study, we enrolled a total of 2035 consecutive newborns admitted to the Neonatal Unit, University Hospital “San Giovanni di Dio e Ruggi d’Aragona,” Salerno, Italy, from November 2022 to April 2025. In detail, we defined the RSV outbreak season, in accordance with the World Health Organization, as the period in temperate climates in which RSV highly circulates, typically in late fall and winter, with the peak lasting for about five months [21]. In Italy, this period is comprised between November and April. For this reason, three consecutive RSV seasons were included in our study: 2024–2025 (nirsevimab introduction); 2023–2024 (pre-nirsevimab); and 2022–2023 (pre-nirsevimab during the COVID-19 pandemic). All infants acquired RSV infection after discharge from the hospital, and only a few cases required NICU admission for severe RSV infection. The inclusion criteria were neonates born or admitted during the study periods, complete clinical records, and parental or guardian consent. The exclusion criteria included missing or incomplete medical records, treatment received outside the study center, and/or lack of consent.

This study adhered to the Declaration of Helsinki and Good Clinical Practice guidelines [22]. In accordance with Italian regulations [23] and the European General Data Protection Regulation (GDPR), Article 89 [24], formal ethical approval was waived for this retrospective analysis of irreversibly anonymized data collected during routine clinical practice, as no additional interventions or data collection occurred. Written informed consent for nirsevimab administration was obtained from parents or guardians as part of the standard clinical practice. The anonymized data were retrospectively extracted from the medical records using a structured CRF, ensuring the absence of personally identifiable information.

### 2.2. Data Collection and Endpoints

The collected data included demographic information (gestational age, weight at birth, sex), medical history (comorbidities, prior respiratory infections), and short-term AEFIs, classified by severity and the time of onset. The newborns were classified as preterm when GA was <37 weeks. Birth weight was categorized as follows: LBW, <2500 g; NBW, 2500–3999 g; and HBW, >4000 g. AEFIs and SAEFIs were classified in accordance with the Italian and European pharmacovigilance guidelines [25,26,27].

The primary endpoints were a season-wide RSV infection rate—defined as laboratory-confirmed cases divided by total newborns in that season—and short-term safety, expressed as the cumulative frequency of any AEFI within 30 days after nirsevimab administration.

### 2.3. Statistical Analysis

Statistical analysis was conducted using SPSS 23.0 and cross-validated using R version 4.4.0. One infant in the 2024–2025 season developed RSV before nirsevimab administration. For the primary season-wide analysis, this infant was included in the numerator and denominator, while the infant was reclassified into the unvaccinated group for all other analyses. The descriptive statistics were expressed as mean ± SD or median (IQR), as appropriate. Season-level RSV infection rates and within-season comparisons between vaccinated and unvaccinated were analyzed using two-sided Fisher exact tests with mid-P correction (Exact 2 × 2 package, R) due to the low number of patients (<20). RR with 95% CI were calculated using the Katz log method. RSV incidence comparisons between seasons were first performed between 2024 and 2025 (nirsevimab), 2023 and 2024 (pre-nirsevimab), 2024 and 2025 and 2022 and 2023 (pre-nirsevimab during the COVID-19 pandemic), or between 2024 and 2025 and pooled pre-nirsevimab seasons, to account for the broader seasonal variation and increase statistical power (exploratory). A within-season 2024–2025 comparison between vaccinated (*n* = 490) and unvaccinated (*n* = 92) infants was also performed. For safety analysis, the exact 95% Clopper–Pearson upper bound was calculated for the AEFI rate. For Bayesian sensitivity analysis, a neutral Beta (1, 1) prior was applied to each season’s infection probability. A total of 100,000 posterior draws were simulated to estimate the probability that the 2024–2025 season had a lower infection rate than either 2023–2024 or the pooled 2022–2024 rate. The results were considered statistically significant if the two-sided *p*-value was <0.05 or the Bayesian posterior probability of benefit was >0.95.

## 3. Results

### 3.1. Safety Profile and Vaccinated Cohort Demographics

In the nirsevimab-exposed cohort (*n* = 491), 53% were male; newborns had a mean GA of 38 ± 2.5 weeks (range, 23–43) and a mean birth weight of 3078 ± 598 g. Preterm neonates (15%) had a mean GA of 34 ± 3.0 weeks (range, 23–36) and a mean birth weight of 2158 ± 651 g (Table 1). First, safety analysis was performed, and no AEFIs occurred among the 491 nirsevimab recipients during the 2024–2025 season (95% upper bound, 0.73%), including term/preterm neonates (Table 1). One infant in the vaccinated cohort received nirsevimab after RSV infection and was included in the overall vaccinated population for demographic and safety descriptions. However, for all efficacy analyses (including infection rates and statistical comparisons), this infant was classified as unvaccinated at the time of infection and counted accordingly.

### 3.2. Season-Wide RSV Incidence

During the 2024–2025 season, in which nirsevimab was introduced into clinical practice, the incidence of RSV infection among the total neonatal population was 0.52%, lower than that observed in the 2023–2024 (1.61%) and 2022–2023 seasons (0.71%) (Table 2). This reduction was slightly significant when compared to the 2023–2024 season (Mid-P exact test, *p* = 0.051), with a corresponding risk ratio of 0.31 for 2024–2025 vs. 2023–2024 (95%CI, 0.09–1.13) and a 69% reduction (Table 2).

Bayesian analysis estimated a 97% probability that RSV infections were lower in the 2024–2025 nirsevimab season compared to 2023–2024. This probability increased to 99% when excluding the infant infected before receiving nirsevimab, while decreased to 89% when compared to pre-nirsevimab seasons (2022–2024), likely due to lower RSV circulation for pandemic-related restrictions (Table 3).

### 3.3. Within-Season Comparison

Within the 2024–2025 cohort, three RSV infections were recorded among a total of 582 newborns. Of these, two occurred in infants who received nirsevimab, resulting in an incidence rate of 0.41% in vaccinated subjects (2/490), while the remaining case occurred in an infant who did not have prophylaxis at the time of infection, corresponding to an incidence of 1.09% among unvaccinated neonates (1/92) (Table 4). Due to the very low number of events, our statistical comparison lacked power and did not reach significance (Mid-P exact test, *p* = 0.40). Nonetheless, the trend suggests a numerically lower infection rate among immunized newborns. One infant who developed RSV prior to nirsevimab administration was classified as unvaccinated for this analysis.

### 3.4. Clinical Outcomes by Season

The clinical severity patterns differed across seasons, although small case numbers precluded statistical comparisons. During nirsevimab implementation (2024–2025), all three hospitalized RSV cases occurred in term infants with distinct outcomes stratified by vaccination status. The single unvaccinated infant developed severe disease requiring 2 days of invasive ventilation, with moderate clinical severity, and bilateral opacities on chest X-ray. In contrast, both nirsevimab recipients experienced markedly milder illness: one required only brief non-invasive respiratory support, while the other one needed no ventilatory assistance, with both showing minimal radiological perihilar changes. Notably, no concurrent infections or NICU admissions occurred in this season’s cohort.

Pre-implementation seasons revealed divergent profiles. In the 2023–2024 season (pre-nirsevimab), complications were more frequent among the reported 12 hospitalizations: two infants required invasive ventilation (mean duration, 3.5 ± 0.7 days), three had documented viral co-infections, two required NICU admission, and four (33%) showed significant radiological involvement including bilateral opacities or diffuse bronchovascular thickening. During the 2022–2023 COVID-19 period, the five hospitalized cases displayed intermediate severity with no invasive ventilation; however, they included one NICU admission, two co-infections (40%), and exclusively mild-to-moderate radiological findings characterized by perihilar or diaphragmatic enhancement. Aggregate clinical metrics showed relative consistency across seasons (Table 5), with a similar mean hospitalization duration (9.7 ± 0.6 days vs. 8.3 ± 2.6 days vs. 9.6 ± 5.4 days) and a mean treatment duration (5.7 ± 1.2 days vs. 6.0 ± 1.7 days vs. 6.4 ± 2.1 days).

Conversely, the proportion of neonates requiring invasive ventilation was higher in 2024–2025 (33%, 1/3) compared to 2023–2024 (17%, 2/12), although the absolute case numbers were minimal and only involved the unvaccinated infant during the immunization season. Vaccination coverage analysis showed that 84.4% (491/582) of eligible neonates received nirsevimab, while 15.6% (91) did not, due to parental reservations.

## 4. Discussion

RSV infection remains a leading cause of morbidity and mortality in newborns and infants, particularly in preterm and those with underlying respiratory conditions. Despite advances in supportive care, effective RSV prevention is still challenging. Nirsevimab, a long-acting MoAb, has shown high efficacy in RSV prophylaxis in newborns. In Italy, its use has been included in routine clinical practice starting from November 2024, thus real-life data on the efficacy and safety of nirsevimab are still poor. In this retrospective, observational, monocentric, real-life pilot study, the efficacy and safety of nirsevimab were investigated in a cohort of term and preterm newborns during the 2024–2025 RSV season. Our findings showed a trend toward lower RSV incidence with nirsevimab introduction compared to the preceding post-COVID-19 2023–2024 season (1.61%). The 2023–2024 reduction is encouraging, as it occurred despite the absence of COVID-19 containment measures that had suppressed RSV circulation during the 2022–2023 season. While this trend aligns with a potential population-level impact of passive immunization strategies, our study design precluded direct causal attribution to nirsevimab administration, and confounding factors could not be excluded.

In our pilot study, we enrolled all newborns admitted to routine care, both term and preterm, during the first season of widespread nirsevimab implementation, unlike controlled trials [28,29,30], which less frequently include preterm infants. Therefore, our results provide a real-world safety assessment in an unselected population of all admitted newborns. Despite this cohort’s heterogeneity, no AEFIs were reported, according to the literature and trials that describe a favorable tolerability profile in neonates [28,31,32,33]. The absence of AEFIs in a real-life setting of unselected newborns, also comprising frail neonates, supports the broader use of this agent, also in near-term infants.

The clinical course of our few RSV cases observed in the 2024–2025 season warrants mention, although the small sample sizes preclude definitive conclusions. All three RSV-positive infants were term neonates, and the single unvaccinated infant developed severe disease requiring invasive ventilation and showed moderate radiological changes. In contrast, the other two infants who developed RSV after nirsevimab experienced milder symptoms, requiring minimal or no ventilatory support, with only mild radiological findings. No co-infections or NICU admissions occurred in this season. While intriguing, this pattern must be interpreted with extreme caution, given the very low event count. Comparatively, RSV cases in the pre-nirsevimab 2023–2024 season were associated with more frequent complications, including invasive ventilation, co-infections, NICU admissions, and more pronounced radiological involvement. Differences in virulence, circulating strains, or clinical management between seasons could contribute to this observed variation in severity, independently from immunization status.

Achieving high coverage remains a challenge, also in our pilot study, as parental reservations accounted for 15.6%, highlighting a significant real-world barrier to maximize the potential population-level benefit of this preventive strategy. In the 2022–2023 season, RSV incidence was low (0.71%) due to the residual effects of the COVID-19 pandemic’s protective measures, such as mask-wearing, hand hygiene, and reduced social mixing. Considering this background is essential when interpreting trends across seasons, as shifting public health behaviors substantially influence infection rates independently of pharmaceutical interventions. Palivizumab was not administered in any infant in the 2024–2025 cohort, thus the safety and incidence reported in our study only reflect the effects of nirsevimab. For the two historical seasons, palivizumab use was undocumented; however, since its indication is limited to very preterm and high-risk infants, its use was likely minimal. Any such use would have biased the earlier-season incidence downward, potentially rendering the apparent benefit of nirsevimab in 2024–2025 a conservative estimate.

This study has several limitations: (i) its single-center, single-arm retrospective design relied on historical rather than contemporaneous controls; (ii) RSV circulation patterns changed during and after the COVID-19 pandemic, and diagnostic intensity likely evolved over time, introducing secular-trend and detection biases; (iii) the exploratory tests were not adjusted for multiplicity, and residual confounding (e.g., GA, comorbidities) could not be excluded; finally, (iv) the very low number of RSV events in our 2024–2025 cohort prevented statistically significant subgroup analyses, even with optimized techniques like mid-P Fisher tests, resulting in underpowered analyses (e.g., 28% power for RR = 0.3). For this reason, we performed a Bayesian sensitivity analysis to contextualize the biological plausibility of a protective effect despite the low absolute case count, revealing a 97% posterior probability of benefit compared to 2023–2024.

A strength of this pilot study is its transparency regarding statistical uncertainty. By applying mid-P adjusted Fisher tests and Bayesian contextualization, we highlighted how near-significant *p*-values still suggest meaningful trends while avoiding overstatement. Furthermore, our power calculations provide essential insights for future research. Using the within-season effect size, we estimate that a future randomized trial would require ≈2300 neonates per group to detect a significant difference with 80% power and *α* = 0.05. For ecological comparisons across seasons, larger samples or multi-season monitoring are needed to account for secular trends, underscoring the necessity of multicenter cohorts.

## 5. Conclusions

In conclusion, this real-world snapshot of neonatal nirsevimab use during its first full season in Italy suggests a favorable safety profile and a potential role in reducing the RSV infection rate. Our findings should be considered hypothesis-generating and provide a foundation for larger, controlled studies of passive immunization in broader newborn populations. Indeed, our results indicate that nirsevimab could be an effective tool for preventing RSV infection in newborns by reducing hospital admissions and severe respiratory syndrome. However, larger, adequately powered randomized controlled trials are needed to confirm its efficacy and safety across diverse clinical settings. In addition, long-term multicenter surveillance is essential to assess the sustained impact of passive immunization and to optimize vaccination strategies.

## Figures and Tables

**Table 1:** Clinical characteristics of newborns immunized with nirsevimab during the 2024–2025 season.

Characteristics	Total Cohort N = 491	Preterm Newborns N = 73	Term Newborns N = 418	*p* Value
M/F, *n* (%)	261 (53)/230 (47)	40 (55)/33 (45)	221 (53)/197 (47)	0.8
Mean GA, weeks (range)	38 ± 2.5 (23–43)	34 ± 3.0 (23–36)	39 ± 1.2 (37–43)	<0.01
LBW, *n* (%)	64 (13)	47 (64)	17 (4)	<0.01
NBW, *n* (%)	415 (85)	26 (36)	389 (93)	
HBW, *n* (%)	12 (2)	0 (0)	12 (3)	
Mean weight at birth ± SD, g	3078 ± 598	2158 ± 651	3238 ± 415	<0.01
Mean LBW weight ± SD, g	1950 ± 482	1786 ± 479	2339 ± 127	<0.01
Mean NBW weight ± SD, g	3223 ± 359	2832 ± 260	3249 ± 350	<0.01
Mean HBW weight ± SD, g	4158 ± 145	-	4158 ± 145	-

**Table 2:** Comparative RSV infection rates across epidemic seasons (2022–2025).

	Season 1 (2024–2025)	Season 2 (2023–2024)	Season 3 (2022–2023)	Season 2 + 3 Combined
Newborns, *n*	582	744	709	1453
RSV infection, *n*	3	12	5	17
Infection rate (95%CI)	0.52 (0.11–1.53)	1.61 (0.84–2.79)	0.71 (0.23–1.64)	1.17 (0.68–1.87)
RR compared to 2024–2025 season *	-	0.31 (0.09–1.13)	1.41 (0.18–3.05)	0.45 (0.13–1.50)
Mid-P Fisher	-	0.051	0.61	0.18
AEFI, *n* (%)	0/491 (0)	-	-	-
95% upper bound	0–0.73	-	-	-

* RR < 1, lower risk in the 2024–2025 season (nirsevimab).

**Table 3:** Bayesian Posterior Probability for Season 1 RSV infection rate.

Comparison	Posterior Probability	Interpretation
2024–2025 vs. 2023–2024	0.97	97% chance that the 2024–2025 season had lower RSV rate
2024–2025 vs. pooled 2022–2024	0.89	89% chance that the 2024–2025 season had lower RSV rate
2024–2025 vs. 2023–2024, (2 cases vs. 12)	0.99	99% chance that the 2024–2025 season had lower RSV rate under stricter analysis

Posterior probability > 95% indicates statistical significance per pre-specified criteria.

**Table 4:** Within-season comparison for 2024–2025.

Group	Total Infants	RSV Cases (*n*)	Infection Rate (%)	RR * (95%CI)	Mid-P Fisher *p*-Value ^$^
Vaccinated	490	2	0.41%	0.38 (0.03–4.1)	0.4
Unvaccinated	92	1	1.09%	-	-

* RR < 1, lower risk in vaccinated group. ^$^, Mid-P Fisher test two-sided, with mid-p correction for small sample.

**Table 5:** RSV clinical outcomes among epidemic seasons.

	2024–2025 (*n* = 3)	2023–2024 (*n* = 12)	2022–2023 (*n* = 5)
Mean age at admission ± SD, days	21 ± 3.6	19.3 ± 5.3	23.4 ± 5.8
Mean hospitalization time ± SD, days	9.7 ± 0.6	8.3 ± 2.6	9.6 ± 5.4
Mean total ventilation support duration ± SD, days	6.3 ± 0.6	6.0 ± 1.7	4.6 ± 1.8
Invasive ventilation, *n* (%)	1 (33%)	2 (17%)	0 (0%)
Mean duration if received, days	0.83 ± 0.83	3.5 ± 0.7	-
Mean treatment duration ± SD, days	5.7 ± 1.2	6.0 ± 1.7	6.4 ± 2.1
Concurrent infections, *n* (%)	0 (0)	3 (25)	2 (40)
NICU Admissions	0	2	1

Posterior probability > 95% indicates statistical significance per pre-specified criteria.

## Data Availability

The data are contained within this article.

## Abbreviations

The following abbreviations are used in this manuscript:

RSV	Respiratory syncytial virus
MoAbs	Monoclonal antibodies
AEFI	Adverse Events Following Immunization
NICU	Neonatal Intensive Care Unit
PNCU	Physiological Neonatal Care Unit
GDPR	General Data Protection Regulation
GA	Gestational age
CRF	Case report form
AEFIs	Adverse events following immunization
LBW	Low birth weight
NBW	Normal birth weight
HBW	High birth weight
SAEFIs	Severe AEFIs
SD	Standard deviation
RR	Relative risks
CI	Confidence interval
